# The Effects of Sprint Interval vs. Continuous Endurance Training on Physiological And Metabolic Adaptations in Young Healthy Adults

**DOI:** 10.2478/hukin-2014-0115

**Published:** 2014-12-30

**Authors:** Gulbin Rudarli Nalcakan

**Affiliations:** 1 Ege University School of Physical Education and Sports, Bornova – Izmir – Turkiye.

**Keywords:** gross efficiency, inflammation, lipids, muscle damage, VO_2_max, Wingate test

## Abstract

The purpose of this study was to compare the effects of sprint interval training (SIT) and continuous endurance training (CET) on selected anthropometric, aerobic, and anaerobic performance indices as well as the blood lipid profile, inflammatory and muscle damage markers in healthy young males. Fifteen recreationally active male volunteers (age: 21.7 ±2.2 years, body mass: 83.0 ±8.0 kg, body height: 1.82 ±0.05 m) were divided into two groups according to their initial VO_2_max levels. Training programs were conducted 3 times per week for 7 weeks. The SIT program consisted of 4–6 Wingate anaerobic sprints with a 4.5 min recovery, while CET consisted of 30–50 min cycling at 60% VO_2_max. Biochemical, anthropometric and fitness assessments were performed both pre and post-intervention. Significant improvements in VO_2_max, anaerobic power and capacity, and VO_2_ utilization during the submaximal workout and significant decreases in body fat and in waist circumference after the intervention occurred in both SIT and CET groups. Significantly greater gross efficiency was measured in the CET group. No differences in the lipid profile or serum levels of inflammatory, myocardial and skeletal muscle damage markers were observed after the training period. The study results agree with the effectiveness of a 30 s all-out training program with a reduced time commitment for anthropometric, aerobic and anaerobic adaptation and eliminate doubts about its safety as a model.

## Introduction

Epidemiological studies have shown that low aerobic capacity is associated with higher rates of cardiovascular diseases, type 2 diabetes mellitus, cancer and mortality (Gist et al., 2014). To lower the disease risk, continuous endurance training (CET) such as running, brisk walking or cycling at moderate intensity is the recommended exercise modality, including 150–250 min•wk-1 activity (Donnelly et al., 2009). As lack of time is a commonly cited reason for physical inactivity, thus, the apparent time efficiency of sprint interval training (SIT) has significant implications for this form of exercise (Burgomaster et al., 2008; Gibala et al., 2006).

It was recently reported that the SIT modality was effective in decreasing subcutaneous fat tissue (Boer et al., 2013), especially in abdominal fat (Boutcher, 2011) and total body weight (Perry et al., 2008), and improving blood lipid and lipoprotein profiles (O’Donovan et al., 2005), aerobic performance and skeletal muscle oxidative capacity (Gibala et al., 2006; Burgomaster et al., 2005), post-exercise fat oxidation and energy consumption (Stepto et al., 2001; King et al., 2002), as well as peak power and mean power output (Bayati et al., 2011; Siahkouhian et al., 2013).

The main ATP resynthesis pathway during CET would be expected to rely on predominantly aerobic energy turnover, whereas SIT relies on anaerobic metabolism. Approximately 15 to 20% of total energy is supplied from oxidative metabolism during a single 30-s Wingate all-out cycling bout (Ozkaya et al., 2014). However, ATP provision derived from oxidative metabolism increases if the exercise bouts are extended (Parolin et al., 1999). The increased contribution from oxidative metabolism during repeated high-intensity effort is attributable to both an increased rate of oxygen transport and utilization and a decreased ability to stimulate ATP production through the breakdown of phosphocreatine and glycogen (Parolin et al., 1999). Nevertheless, it appears that SIT induces the same physiological and metabolic adaptations as traditional CET. These muscle adaptations have been associated with improved glycemic control and insulin sensitivity (Sloth et al., 2013). In contrast, the improvements observed in muscular oxidative capacity are related to work loads and fluctuations in oxygen uptake during exercise rather than to exercise duration and energy consumption (Daussin et al., 2008).

Although health- and fitness-related benefits of SIT have been demonstrated, it is premature to recommend the SIT modality, particularly to a population with cardiovascular disease as well as older and sedentary individuals (Whyte et al., 2010). Although SIT might be considered an appropriate physical activity option for healthy young people, intense training induces impaired immune responses (Robson-Ansley et al., 2007), increased skeletal muscle damage (Clarkson and Hubal, 2002) and myocardial cell injury (Rogers et al., 1985; Vidotto et al., 2005). However, it is unknown whether this form of exercise can be tolerated by healthy young people whose fitness level is low.

Although currently available literature has focused on the basic fitness effects of the allout sprint interval modality, there is a lack of experimental studies that evaluate SIT effects vs. CET based on fitness, general health and safety aspects considered together. Therefore, the aim of this study was to compare the effects of SIT and CET on anthropometric, aerobic, anaerobic performance indices, mechanical gross efficiency, blood lipids, inflammation, skeletal muscle damage and myocardial cell injury in healthy young males. It was hypothesized that similar anthropometric, physiological and biochemical progress and adaptation would be observed in SIT and CET.

## Material and Methods

### Participants

Fifteen healthy young recreationally active university students (mean ± standard deviation; age: 21.7 ±2.2 years, body mass index (BMI): 25.0 ±2.1 kg·m^−2^, percentage of body fat (BF%): 16.2 ±3.2%, VO_2_max: 40.3 ±5.0 ml·min^−1^·kg^−1^) volunteered to participate in this investigation. All participants were habitually active but not engaged in any sort of structured training program, nor had they been so engaged for at least 5 months prior to the study. They were instructed not to perform additional exercise, modify their regular daily diet regimen, or take any medications or supplements during the study period. After routine medical screening, the subjects were informed of the procedures to be employed in the study and associated risks, and all provided written informed consent. The experimental protocol was approved by the Local Scientific Research Ethics Board (approval no: 20.478.486–278).

### Experimental Design

A repeated measures study design was used for this prospective laboratory experiment. Prior to any baseline testing, all participants visited the laboratory for fasting blood samples and body composition measurements. The following day, they participated in a familiarization session to minimize any learning effect and to adapt to the testing/training procedures, the laboratory environment and our study group. Before the training periods, a submaximal test, a maximal graded exercise test and then a verification phase were performed to determine the initial VO_2_max levels of the volunteers. A 30-second Wingate all-out test was conducted to measure anaerobic indices. A 30-min constant-loading submaximal test was then performed. All these tests were separated by intervals of at least 24 hours. Subjects were matched according to their initial VO_2_max levels and divided into two groups as follows (numbers indicate ranking of VO_2_max values; 1= the highest, 15= the lowest):

**Table:** 

CET:	1	4	5	8	9	12	13	
SIT:	2	3	6	7	10	11	14	15

After the baseline measurements were completed, the subjects began their 7-week sprint interval or continuous endurance training. In the middle of the training period, a graded exercise test was performed to reregulate the training loads. At the end of the training period, the baseline tests were repeated in the same order. Standard environmental conditions (20–23 ºC ambient temperature, 55–65% relative humidity) were ensured during the testing sessions.

### Anthropometric Measurements

The anthropometric measurements were performed by the same researcher on all subjects according to the Anthropometric Standardization Reference Manual (Lohman et al., 1988). The BMI was calculated from height and body mass with the formula body mass (kg) / height (m^2^). All subcutaneous skinfold measurements were taken with a Holtain skinfold caliper (Holtain Ltd., UK) in triplicate. BF% was calculated using the Faulkner equation (Martínez et al., 2011). The sum of eight skinfolds (SS) (thicknesses of the triceps, biceps, chest, subscapula, suprailiac, abdominal, thigh and calf) was also evaluated. Waist circumference was measured midway between the lower rib margin and iliac crest. Hip circumference was measured at the level of widest circumference over the greater trochanters. The waist-to-hip ratio (W/H) was calculated as waist circumference divided by hip circumference.

### Physiological Measurements

Oxygen uptake was measured breath-by-breath using a Cosmed Quark b2 (Cosmed Srl, Rome, Italy) with expired gas concentrations. The Quark b2 was calibrated according to the manufacturer’s instructions. The turbine flow meter was calibrated using a 3-L syringe (Quinton Instruments, USA). HR data were collected with a system compatible with the same gas analyzer (Polar Electro OY, Kempele, Finland).

### Familiarization Sessions

The participants visited the laboratory before the main study. The aim of this visit was to adapt the participants to the laboratory environment and staff and, in addition, to minimize the learning effect and to familiarize the participants with the testing/training procedures. The familiarization sessions for the submaximal and maximal graded exercise tests consisted of four 5-min stages. The first stage was initiated with a workload of 60–80 Watts and continued in ∼10 Watt increases. To become familiar with the Wingate all-out test procedures, the participants performed a Wingate test trial. All procedures were performed on a mechanically braked cycle ergometer (894E, Monark, Sweden) which was set up to replicate the participant’s normal riding position for all tests.

### Submaximal Tests

The purpose of this test was to determine the initial load for the maximal graded exercise test. Therefore, the submaximal test consisted of four 5-min stages in which load increments were included to keep the subjects within the limits of the predicted aerobic threshold (heartbeat of 130–150 bpm) and anaerobic threshold (heartbeat of 150–170 bpm). Respiratory exchange dynamics were also considered in accordance with the VE/VO_2_. The cadence was fixed at 60 rpm for the test.

### Maximal Graded Exercise Tests and Verification Phases

The initial loads of the graded VO_2_max test were adjusted to the respiratory anaerobic threshold level predicted from the submaximal test. The test focused on voluntary exhaustion by 10±2 min and was fixed at 60 rpm. Accordingly, the test load was increased by ∼20 Watts at the 4th, 6th, 8th, 10th, 11th and 12th min. VO_2_max was calculated as the highest value of oxygen uptake observed over a 30-s period. VO_2_max was confirmed when three or more of the following criteria were met: (1) a plateau in VO_2_ despite an increase in the work load, (2) a respiratory exchange ratio (RER) higher than 1.2, (3) a peak heart rate at least equal to 90% of the age-predicted maximum, and/or (4) visible exhaustion. The subjects were given strong verbal encouragement to exercise until volitional exhaustion but were not given progress feedback. The purpose of the verification test was to confirm the VO_2_max level. This test started with the last-stage load of the incremental test and continued until voluntary exhaustion occurred between the 4th and 7th min of the test.

### Constant-Loading Submaximal Tests

The 30-min exercises were completed at the load corresponding to 60% of the VO_2_max. The mechanical gross efficiency (GE%) was calculated to show the metabolic efficiency in the constant-loading submaximal tests. Metabolic rate (MR) was calculated with the formula given by Nishi (1981), MR (W) = 21× (0.23 × RER + 0.77) × (VO_2_)^−1^, where GE is defined as the measured mechanical power output divided by the calculated MR.

### Wingate All-Out Test

The participants performed a standardized 5-min warm up prior to experimental data collection and performed the test against a resistance equivalent to 7.5% of the subject’s body mass. They were instructed to pedal as rapidly as possible against the ergometer’s inertial resistance. The appropriate load was then applied to allow the participants to start at 120 rpm, and data capture was initiated. The subjects were verbally encouraged to pedal continuously throughout the 30-s test. Peak power (PP), average power (AP), power drop (PD), the fatigue index (FI) and time to reach PP (tPP) were then calculated over the Wingate test. The FI (%) was determined over the mean power data for the 5-s intervals, whereas the PD (%) was calculated based on the instantaneous power data obtained with millisecond sensitivity.

### Biochemical Measurements

Fasting blood samples were collected in the morning (08.00–10.00 a.m.) by the same investigator and collected from an antecubital forearm vein, then centrifuged at 4000 rpm for 10 min, extracted and placed into plastic storage vacutainers to attempt to control for the lipid profile, inflammation, myocardial and skeletal muscle damage. White blood cell (WBC) levels were analyzed in an automated COULTER® LH 780 Hematology Analyzer, whereas analyses of cardiac troponin I (cTnI) levels were performed by an automated chemiluminescence system (Beckman Coulter Access 2). Creatine kinase (CK), creatine kinase-myocardial band (CK-MB), aspartate aminotransferase (AST), alanine aminotransferase (ALT), C-reactive protein (CRP), total cholesterol (TC), very low-density lipoprotein (VLDL), high-density lipoprotein (HDL), and triglyceride (TG) levels were quantified in an automated Beckman Coulter Olympus Au 2700 chemical analyzer. Low-density lipoprotein (LDL) content was calculated from the Friedewald formula.

### Training Intervention

The SIT program consisted of 4–6 Wingate anaerobic sprints with a 4.5-min recovery, and the CET program consisted of 30–50 min of cycling at 60% of VO_2_max. The loads, repetitions for SIT and time for CET were gradually increased during the training periods. Training was performed three times per week for seven consecutive weeks. A rest of 1–2 days between sessions was provided to promote recovery.

### Statistical analysis

Descriptive statistics were reported as the mean ± SD. The Shapiro–Wilk *W* test and Levene’s test, respectively, showed that the data obtained met the assumptions of normality and homogeneity of variance. Therefore, parametric statistical tests were used. Group (CET and SIT) was the between-subjects factor, and Time (0. Week and 7. Week) was the within-subjects factor in the present study. The main effects and the interaction effect of these factors on the dependent variables were assessed using a 2 × 2 (Group × Time) two-factor mixed-design analysis of variance (ANOVA). Aerobic power indices were measured at three different time points (0, Week 3, Week 7) across the study. Therefore, a 2 × 3 (Group × Time) two-factor mixed-design ANOVA was used in the analysis of these variables. In case of a significant Time effect or Group × Time interaction effect, a one-factor repeated measures ANOVA with the Bonferroni post-hoc procedure was performed to identify the source of the difference. These tests were also performed in case of insignificant overall mixed-design ANOVA results to identify the effect size (d) of the difference, which is an important determinant of practical significance. The effect size of the difference was evaluated using the classification of Cohen (<0.2 trivial, 0.2 ≤d<0.5 small, 0.5 ≤d<0.8 moderate, d≥0.8 large effect size). Baseline values of dependent variables were compared between CET and SIT using an independent-samples t-test. The level of statistical significance was set at p ≤ 0.05.

## Results

Because of health problems and the intensive test program, three participants failed to continue the experimental study. As a result, the experimental phase was completed by 15 participants. After the performance of 21 sessions over 7 weeks, waist and hip circumference, BF% and SS in the CET, waist circumference, W/H, BF% and SS in the SIT significantly decreased. There were no significant differences between CET and SIT in anthropometric results (Table 1)

Statistically significant main effects for time were detected for BF (p<0.001, ηp2=0.057), SS (p<0.001, ηp2=0.643), VO_2_max (p<0.001, ηp2=0.698), PP (p<0.001, ηp2=0.669), AP (p<0.001, ηp2=0.729), FI (p=0.027, ηp2=0.324), tPP (p<0.016, ηp2=0.371), GE (p<0.001, ηp2=0.645), and cTnI (p<0.021, ηp2=0.348). However, no significant Group × Time interaction effect was found for any of the dependent variables, indicating that changes in these variables over the course of the study showed similar patterns in the CET and SIT groups.

Among the aerobic fitness variables, there were significant differences for maximal oxygen uptake (VO_2_max) values at pre, mid and post-test (W0–W3–W7) in the CET group, and there were also significant improvements among the VO_2_max values at pre, mid and post-test (W0–W3–W7), except between W0 and W3, in the SIT group. VO_2_max improvement was similar in the CET (8.7%) and SIT (7.0%) groups. There were no significant differences between CET and SIT in the indices of the aerobic power test (Table 2).

Table 3 shows that there were significant differences between the pre and post-test PP (W/kg) results between groups (0.008 and 0.011, respectively). After the 7-week training periods, only PP (W·kg-1) and AP (W·kg-1) increased significantly in the CET and SIT groups.

To calculate the GE (%), the participants performed a 30-min submaximal test (60% of VO_2_max) with constant loading. According to the test results, oxygen uptake per liter (VO_2_) was significantly increased in the CET and SIT groups, whereas GE% was significantly improved only in the CET group. No differences in these parameters were found between groups (Table 4).

Our results showed that the type and duration of training had no significant effect on the cholesterol profile (LDL, HDL, VLDL, TC, TG), serum levels of inflammatory markers (CRP, WBC), myocardial cell injury markers (cTnI and CK-MB) or skeletal muscle damage markers (CK, ALT, and AST) (p>0.05). The levels of the CRP and WBC counts differed between the two training groups. Although they increased (9.7%, 9.8%) after SIT and decreased (2%, 26%) for WBC and CRP after CET, these differences were not statistically significant (Tables 5 and 6).

## Discussion

Although many exercise programs prescribed for fat reduction involve continuous and moderate aerobic exercise, the SIT modality may be as successful as traditional methods for fat loss. Indeed, it has been shown that SIT may be preferable for reducing body fat (Hazell et al., 2014; Macpherson et al., 2011) and decreasing waist and hip circumference (Whyte et al., 2010). The results of the present study demonstrated significant decreases in waist circumference, the waist-to-hip ratio and body fat in SIT, with similar results found for the CET group. These results support the hypothesis that increased post-exercise VO_2_, fat oxidation, reduced RER and carbohydrate oxidation after a single SIT bout (Whyte et al., 2010; Hazell et al., 2012) may be significantly related to fat loss. Moreover, the energy cost and VO_2_ during exercise are relatively low (Hazell et al., 2012; Tremblay et al., 1994). It is known that a decreased waist circumference, as reflected by a reduced abdominal fat mass and the waist-to-hip ratio, is particularly important because of the inverse relationship between abdominal fat and health status.

Although SIT has been used to improve anaerobic performance (Whyte et al., 2010; MacDougall et al., 1998), this exercise modality has recently gained popularity as a means of enhancing aerobic status in only a few sessions (Burgomaster et al., 2005; Burgomaster et al., 2006). The results of the present study showed similar benefits from SIT and from traditional CET (Burgomaster et al., 2008; Gibala et al., 2006; Sandvei et al., 2012). The effectiveness of SIT may stem from a combination of anaerobic and aerobic energy demands. Although the anaerobic contribution is dominant for the first 30 s all-out bout, the aerobic contribution gradually increases throughout repeated Wingate bouts (Parolin et al., 1999). Gist et al. (2014) have shown that adaptations include rapid increases in muscle oxidative capacity, as reflected by key mitochondrial enzymes, glucose transporters and muscle membrane lactate transporters.

Moreover, aside from changes in anthropometric, aerobic and anaerobic performance indices, the SIT modality may be preferred because of its greater time efficiency compared with CET. Indeed, SIT is a time-efficient strategy for both performance-based programs and commercially available fitness planning purposes. A total of 8.45 hours (only 52.30 min actively) was spent in the SIT modality, whereas a total of 14 hours was spent in CET during the seven-week training period in the present study.

There is no evidence that sprint training produces an increase in GE during submaximal exercise. However, GE is accepted as a key for peak performance for racing distances of 5000 meters and more. It is known that efficiency is a measure of effective work and is most commonly expressed as the percentage of total energy expended that produces external work. The GE of the human body is generally in the range of 10–25% during submaximal cycle exercise (Moseley and Jeukendrup, 2001). Although GE improvement was significant in CET, it was not significant in the SIT group (18% vs. 9%). These results may imply that the CET modality induces peripheral adaptations in preference to central adjustments. Conversely, SIT appears to induce central adaptations, as evidenced by stroke volume improvement (Trilk et al., 2011).

After exhaustive endurance exercise, muscle damage can be produced by metabolic disturbances associated with ischemia. An increased CK level in the blood after intense exercise may be interpreted as indicating possible mechanical injury to the musculoskeletal system due to the production of an inflammatory response (Toft et al., 2002), but regular training is known to reduce muscle damage caused by exercise (Jaffe et al., 1984), and repeated high doses of exercise at sufficient intervals are known to cause relatively less muscle damage. Priest et al. (1982) confirmed a positive correlation between training status and resting CK and CK-MB levels. The serum AST, ALT, CK and CK-MB activity levels in the SIT group were higher than those in the CET group, indicating intensive exercise-caused muscle cell damage at higher rates in the present study. The increased CK-MB level in skeletal muscle was perhaps due to increases in satellite cells, which repair injured skeletal muscle (Jaffe et al., 1984). Because CK-MB did not increase above 4.5% as a percentage of total CK, the increase in this isoenzyme originated from skeletal muscle rather than cardiac muscle. Moreover, cTnI did not show a statistically significant increase following SIT.

Several studies have been performed to investigate the effects of exercise on cardiac troponins (cTn), which are among the newer cardiac markers; in particular, cTnI can be found in slow and fast-twitch muscles as well as in the myocardium (Davies et al., 1997). It can be accepted that mild increases in serum cTn often follow prolonged endurance exercise (Vidotto et al., 2005), despite contradictions in the literature. These conflicts may result from differences in the fitness levels of participants, the type or duration of exercise, timing of the post-exercise sample, the troponin assay used, and the detection limit used to define a “positive” cTn. Although previous findings strongly suggest that exercise intensity and duration are important determinants of circulating post-exercise cTn, further laboratory-based assessments are needed to clarify this issue (Shave et al., 2010).

It is known that leukocyte count and inflammation markers increase following intense interval training (Robson-Ansley et al., 2007; Markovitch et al., 2008), as increases in inflammatory mediators may indicate their role in muscle tissue repair after anaerobic exercise. However, they have been found to be markedly reduced in skeletal muscle after regular endurance training (Hovanloo et al., 2013). These results are in line with our findings that serum levels of CRP decreased in CET (26%) and that WBC and CRP increased in SIT (9.7% and 9.8%, respectively), but these differences were not statistically significant.

Studies focused on blood lipid changes in exercise programs of different intensities have obtained controversial results. It has been suggested that high intensities are not required if changes in lipids are the goal of training (Crouse et al., 1997); however, it has been reported that changes in lipids are influenced by exercise intensity (O’Donovan et al., 2005). It has been found that SIT is more effective than CET for improving cholesterol profiles in healthy subjects (Sandvei et al., 2012). Although several studies have focused on the aerobic effects of SIT, knowledge is lacking about the effects of the SIT modality on blood lipid levels. The findings of the current study are consistent with the study of Crouse et al. (1997), which showed no significant effect of exercise intensity on the lipid profile over a course of 24-week cycle ergometer training (Crouse et al., 1997). It should be considered that conclusions drawn from several of the published studies are limited by the absence of a control group, differences among studies in mode and volume of training, subject pre-training lipid levels, diet control, and genetic basis for the elevated cholesterol, as well as by a failure to control for the acute effects of the most recent training session (Crouse et al., 1997).

This study demonstrated that seven weeks of SIT improved body composition, aerobic and anaerobic capacity to an extent that was statistically similar to the corresponding results of CET. It appears that no differences occurred for the lipid profile, serum levels of inflammatory markers, myocardial cell injury markers and skeletal muscle damage markers after any training period. The SIT protocol can be recommended as a time-efficient strategy after a moderate-intensity training period.

The major limitation of the current study is the relatively small sample size and the absence of control of the caloric intake of the subjects. Therefore, the results of this study should be considered in terms of the assumption that each subject followed his regular daily diet regimen according to the instructions of the researcher. Further studies that will be conducted on large samples with strict control of caloric intake will provide more reliable information about the SIT modality for the relevant areas of study.

## Figures and Tables

**Figure 1:**
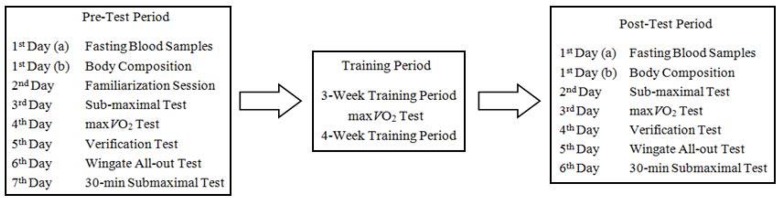
Flow chart of the study protocol

**Table 1:** Influence of CET and SIT on anthropometric indices

	CET (n=7)						SIT (n=8)					
	
	Week No	M ± SD	Δ%	p	d	Week No	M ± SD	Δ%	p	d
mass (kg)	W0	79.3 ± 6.69	−1.1	0.140	−0.132	W0	86.2 ± 8.00	−0.6	0.530	−0.065
	W7	78.4 ± 6.96				W7	85.7 ± 7.42			
BMI (kg·m ^−2^)	W0	24.5 ± 1.90	−1.2	0.137	−0.154	W0	25.5 ± 2.23	−0.8	0.573	−0.090
	W7	24.2 ± 2.00				W7	25.3 ± 2.20			
waist (cm)	W0	82.4 ± 3.94	−3.9	0.001^*^	−0.868	W0	86.8 ± 7.88	−3.3	0.023^*^	−0.393
	W7	79.2 ± 3.41				W7	83.9 ± 6.85			
hip (cm)	W0	100 ± 4.86	−4.0	0.002^*^	−0.711	W0	102 ± 4.11	−0.1	0.479	0.239
	W7	96.0 ± 6.30				W7	101 ± 4.24			
W/H (%)	W0	0.82 ± 0.31	1,2	0.655	0.030	W0	0.85 ± 0.48	−2.4	0.033^*^	−0.040
	W7	0.83 ± 0.36				W7	0.83 ± 0.51			
BF (%)	W0	15.8 ± 2.62	−5.7	0.015^*^	−0.343	W0	16.5 ± 3.72	−7.3	0.007^*^	−0.348
	W7	14.9 ± 2.63				W7	15.3 ± 3.15			
SS (mm)	W0	114 ± 24.3	−10.5	0.015^*^	−0.470	W0	124 ± 43.1	−10.5	0.010^*^	−0.327
	W7	102 ± 26.7				W7	111 ± 36.0			

* p≤0.05,

CET: continuous endurance training, SIT: sprint interval training, BMI: body mass index, W/H: waist-to-hip ratio, BF: body fat, SS: sum of 8 skinfolds, M: mean, SD: standard deviation, Δ%: percentage change in means, d: Cohen’s d (<0.2 trivial; 0.2 ≤ d <0.5 small; 0.5 ≤ d <0.8 moderate; d ≥ 0.8 large effect size), W: week

**Table 2:** Influence of CET and SIT on indices of the aerobic power test

	CET (n=7)						SIT (n=8)					

	Week No	M ± SD	Paired Weeks	Δ%	p	d	Week No	M ± SD	Paired Weeks	Δ%	p	d
VO_2 max_ (ml•min−1•kg−1)	W0	40.5 ± 6.0	W3-W0	4.4	0.038^*^	0.327	W0	40.2 ± 4.3	W3-W0	3.0	0.099	0.292
	W3	42.3 ± 5.0	W7-W3	4.1	0.018^*^	0.358	W3	41.4 ± 3.9	W7-W3	3.9	0.009^*^	0.410
	W7	44.0 ± 4.8	W7-W0	8.7	0.014^*^	0.654	W7	43.0 ± 3.9	W7-W0	7.0	0.022^*^	0.682

* p≤0.05,

CET: continuous endurance training, SIT: sprint interval training, VO_2_max: maximal oxygen consumption, M: mean, SD: standard deviation, Δ%: percentage change in means, d: Cohen’s d (< 0.2 trivial; 0.2 ≤ d < 0.5 small; 0.5 ≤ d < 0.8 moderate; d ≥ 0.8 large effect size), W: week

**Table 3:** Influence of CET and SIT on indices of the Wingate all-out test

	CET (n=7)					SIT (n=8)				

	Week No	M ± SD	Δ%	p	d	Week No	M ± SD	Δ%	p	d
PP (W·kg^−1^)	W0	11.4 ± 1.4	9.6	0.008^*^	0.935	W0	12.3 ± 1.7^ǂ^	8.9	0.011^*^	0.686
	W7	12.5 ± 0.9				W7	13.4 ± 1.5^#^			
AP (W·kg^−1^)	W0	8.10 ± 0.50	3.7	0.033^*^	0.663	W0	8.28 ± 0.70	3.9	<0.001^*^	0.457
	W7	8.40 ± 0.40				W7	8.60 ± 0.70			
FI (%)	W0	44.0 ± 10.1	18.6	0.122	0.816	W0	51.2 ± 6.0	6.4	0.091	0.602
	W7	52.2 ± 10.0				W7	54.5 ± 4.9			
tPP (s)	W0	1.94 ± 0.73	−34.5	0.074	−1.195	W0	1.39 ± 0.37	−12.3	0.092	−0.559
	W7	1.27 ± 0.31				W7	1.22 ± 0.22			
PD (%)	W0	57.0 ± 8.9	9.8	0.086	0.636	W0	64.2 ± 6.8	0.8	0.811	0.083
	W7	62.6 ± 8.7				W7	64.7 ± 5.1			

* p≤0.05,

ǂ p=0.040,

# p=0.004 (statistically significantly higher compared with data of CET), CET: continuous endurance training, SIT: sprint interval training, PP: peak power, AP: average power, FI: fatigue index, tPP: time to peak power, PD: power drop, M: mean, SD: standard deviation, Δ%: percentage change in means, d: Cohen’s d (<0.2 trivial; 0.2 ≤ d < 0.5 small; 0.5 ≤ d < 0.8 moderate; d ≥ 0.8 large effect size), W: week

**Table 4:** Influence of CET and SIT on indices of gross efficiency

	CET (n=7)					SIT (n=8)				

	Week No	M ± SD	Δ%	p	d	Week No	M ± SD	Δ%	p	d
*V*O_2_ (L)	W0	1.96 ± 0.34	12.8	0.011^*^	0.826	W0	1.97 ± 0.24	18.3	0.001^*^	1.765
	W7	2.21 ± 0.26				W7	2.33 ± 0.16			
GE (%)	W0	16.3 ± 1.59	18.4	0.001^*^	1.558	W0	17.3 ± 2.43	8.8	0.071	0.800
	W7	19.3 ± 2.21				W7	18.8 ± 1.19			

* p≤0.05,

CET: continuous endurance training, SIT: sprint interval training, GE: gross efficiency, VO_2_: oxygen consumption, M: mean, SD: standard deviation, Δ%: percentage change in means, d: Cohen’s d (<0.2 trivial; 0.2 ≤ d < 0.5 small; 0.5 ≤ d ≤ 0.8 moderate; d > 0.8 large effect size), W: week

**Table 5:** Influence of CET and SIT on indices of the lipid profile

	CET (n=7)					SIT (n=8)				

	Week No	M ± SD	Δ%	p	D	Week No	M ± SD	Δ%	p	d
TC (mg/dl)	W0	164 ± 26.8	1.2	0.749	0.086	W0	178 ± 31.4	−1.1	0.817	−0.057
	W7	166 ± 18.9				W7	176 ± 38.8			
HDL (mg/dl)	W0	48.1 ± 5.60	−1.5	0.747	−0.125	W0	51.0 ± 10.7	2.9	0.111	0.142
	W7	47.4 ± 5.60				W7	52.5 ± 10.5			
LDL (mg/dl)	W0	98.6 ± 22.1	0.8	0.813	0.039	W0	104 ± 27.9	6.7	0.345	0.247
	W7	99.4 ± 18.8				W7	111 ± 29			
VLDL (mg/dl)	W0	17.1 ± 10.2	9.4	0.368	0.135	W0	23.0 ± 7.50	−6.1	0.562	−0.166
	W7	18.7 ± 13.3				W7	21.6 ± 9.30			
TG (mg/dl)	W0	86.0 ± 51.4	8.1	0.420	0.117	W0	115 ± 38.1	−6.1	0.543	−0.166
	W7	93.0 ± 67.0				W7	108 ± 45.9			

CET: continuous endurance training, SIT: sprint interval training, TC: total cholesterol, HDL: high-density lipoprotein, LDL: low-density lipoprotein, VLDL: very low-density lipoprotein, TG: triglyceride, M: mean, SD: standard deviation, Δ%: percentage change in means, d: Cohen’s d (<0.2 trivial; 0.2 ≤ d < 0.5 small; 0.5 ≤ d < 0.8 moderate; d ≥ 0.8 large effect size), W: week

**Table 6:** Influence of CET and SIT on indices of inflammatory effect, myocardial damage and skeletal muscle damage

	CET (n=7)					SIT (n=8)				

	Week No	M ± SD	Δ%	p	D	Week No	M ± SD	Δ%	p	d
WBC (10^3^/µL)	W0	7.92 ± 2.35	−2.1	0.758	−0.078	W0	6.91 ± 1.53	9.7	0.321	0.506
	W7	7.75 ± 2.02				W7	7.58 ± 1.08			
CRP (mg/L)	W0	4.39 ± 2.76	−26.2	0.312	−0.589	W0	3.27 ± 0.24	9.8	0.475	0.388
	W7	3.24 ± 0.13				W7	3.59 ± 1.14			
AST (U/L)	W0	15.9 ± 4.81	0.0	1.000	0.000	W0	17.4 ± 4.37	12.9	0.227	0.489
	W7	15.9 ± 2.41				W7	19.6 ± 4.78			
ALT (U/L)	W0	11.1 ± 2.79	15.4	0.373	0.652	W0	16.4 ± 8.95	34.3	0.018^*^	0.741
	W7	12.9 ± 2.48				W7	22.0 ± 5.92^ǂ^			
CK (U/L)	W0	132 ± 61.1	11.5	0.743	0.217	W0	134 ± 40.3	31.1	0.447	0.378
	W7	147 ± 77.4				W7	175 ± 150			
CK-MB (U/L)	W0	13.5 ± 5.17	12.9	0.557	0.327	W0	12.8 ± 4.46	20.0	0.320	0.639
	W7	15.3 ± 5.53				W7	15.4 ± 3.53			
cTnI (ng/mL)	W0	0.031 ± 0.079	16.1	0.078	0.062	W0	0.000 ± 0.000	0.3	0.170	0.849
	W7	0.036 ± 0.081				W7	0.003 ± 0.005			

ǂ p=0.002 (statistically significantly higher compared with data of CET), CET: continuous endurance training, SIT: sprint interval training, WBC: white blood cell, CRP: C-reactive protein, AST: aspartate aminotransferase, ALT: alanine aminotransferase, CK: creatine kinase, CK-MB: creatine kinase-myocardial band, cTnI: cardiac troponin I, M: mean, SD: standard deviation, Δ%: percentage change in means, d: Cohen’s d (<0.2 trivial; 0.2 ≤ d < 0.5 small; 0.5 ≤ d ≤ 0.8 moderate; d > 0.8 large effect size), W: week
